# Supplementary material for: Identification of GLI1 and KIAA0825 Variants in Two Families with Postaxial Polydactyly
Source: Genes (Basel). 2023 Apr 5;14(4):869. doi: 10.3390/genes14040869 (PMC10137575; doi:10.3390/genes14040869)
Supplement: Supplementary file 1 [file genes-14-00869-s001.zip › genes-2295948-supplementary.pdf]

Table S1. Protein-Protein interaction of the wildtype and mutant KIAA0825 with C1ORF167 and the wildtype and mutant GLI1 with SUFU

**KIAA0825-C1orf167 Interaction**				
**Interacting portein**	**Wildtype KIAA0825 Interacting residues**	**Mutant (p.Pro1191Leu) KIAA0825 Interacting residues**	**Close Interactor C1orf167 residues**	**Number & type of Bond**
Wildtype KIAA0825-C1orf167	Lys105, Glu107, Gln187, Ser246	________	Ser1412, Lys1422, Glu1425, Glu1429,His1308	3 hydrogen bonds & 2 salt bridges
Mutant (p.Pro1191Leu) KIAA0825-C1orf167	________	Gln864, Glu875, Arg925, Tyr950, Glu1027, Asp1028,Glu1078, Asn1271	Arg4, Lys16, Arg111, Lys114, Gln466, Val833, Gln971, Gly1015, Arg1432	7 hydrogen bonds & 2 salt bridges
**GLI1-SUFU Interaction**				
**Interacting proteins**	**Wildtype GLI1 Interacting residues**	**Mutant (p.Arg113*) GLI1 Interacting residues**	**Close Interactor SUFU residues**	**Number & type of Bonds**
Wildtype GLI1-SUFU Protein Interaction	Asp347, Gly526, His983, Pro 989, Arg990	________	Asp47, Arg75, Glu86, Glu127, Gly241	3 hydrogen bonds & 3 salt bridges
Mutant (p.Arg113*) GLI1-SUFU Protein Interaction	________	Asn45, Ser92, Val98, Ser112	Thr13, Asp47, Gln53,Pro133	4Hydrogen bonds
